# Health-related Quality of Life of Nurses Working in Tertiary Care Hospital of Karachi

**DOI:** 10.12669/pjms.36.3.1267

**Published:** 2020

**Authors:** Amjad Ali, Abdur Rasheed, Subia Naz

**Affiliations:** 1 Mr. Amjad Ali, MS-Nursing. Lecturer, Institute of Nursing, Dow University of Health Sciences, Karachi, Pakistan; 2 Dr. Abdur Rasheed, PhD Statistic. Assistant Professor, School of Public Health, Dow University of Health Sciences, Karachi, Pakistan; 3 Ms. Subia Naz, Lecturer, Institute of Nursing MS-Nursing, Dow University of Health Sciences, Karachi, Pakistan

**Keywords:** Depression, Health, Nurses, Health-related quality of life

## Abstract

**Objective:**

The aim of this study was to assess nurses’ health-related quality of life.

**Methods:**

This cross-sectional analytical study was accomplished among 154 nurses. Data were collected from two tertiary care hospitals of Karachi. Consent was taken prior to data collection from every participant. Questionnaire Short Form Health Survey-26 (SF-26) and Patients Health Questionnaire-9(PHQ-9) were used as a study tool. Data were analyzed through SPSS version 21.

**Results:**

ANOVA and T-test confirmed that energy/fatigue domain differed significantly with level of education, duty shift and monthly income with p-values 0.025, 0.001 and 0.006 respectively. It was observed that mean scores of physical functioning, role limitation due to physical health and pain domains differ significantly between depressive and non-depressive participants, with p-values 0.045, 0.01 and 0.005 respectively.

**Conclusions:**

Health related quality of life differs in comparison of physical health domain with depressive and non-depressive nurses. Only energy/fatigue domain was significantly associated with level of education, duty shift and monthly income of nurses.

## INTRODUCTION

The health-related quality of life (HRQoL) was initiated in the field of medical sciences, focusing primarily on the estimation of physical and psychological condition and social well-being.^1^ Still, there is no precise meaning of the HRQoL that has been established by all researchers. In its broadest sense, it is characterized as a quality of life area focusing mainly on health assessment.^2^

Health-related quality of life (HRQoL) estimation has increasingly been recognized as important for decision making in the community and clinical settings; it gives information about the performance and wellbeing of population.^3^ HRQoL is quickly gaining recognition as a quantifiable outcome. It’s a wide multidimensional idea that naturally encompasses self –reported assessment of psychological state, functional capability, social function, and individual awareness of own health.^4^

Nurses’ everyday life practices have significant consequences for their health consequences that can increase the risk of physical and psychological health problems. Additionally, nurses’ health and wellbeing may directly link to the patient’s quality care and health of the population.^5^

Globally, studies on nurses’ health have to pay attention primarily on the performance of nurses in community health settings whereas very few exploratory studies have investigated the health of nurses related to hospital setting.^6^ Most of the studies focused on nurses’ clinical skills related to the emphasis on patients care, but a few studies have identified the personal lifestyle.^7^

The studies show the vitality of nurses’ good health due to its direct impact on patients care.^8^ Quality of care and patient safety can be compromised due to the poor health status of nurses.^9^ Some studies have also reported the link between the poor health of nurses with medication errors.^9^,^10^ Oyama and Fukahori highlighted that the assessment of nurses’ health can guide to generate a policy for maintaining a healthy working environment.^9^

Globally, psychological problems are increasing day by day, especially anxiety and depression. Both problems linked to stressful conditions. Additionally, depression often comes with symptoms of anxiety.^11^ Approximately 350 million people with different age group were suffering from depression globally. More than 0.8 million people suicide related to depression globally.^12^ It was observed that young adult and adolescents suffering from anxiety, depression, and stress is from 5% to 70% of range throughout the world.^13^

Nurses are considered as a backbone of the health care system. Nurses have busy schedule to perform duties in different departments of an organization and may be exposed to the chemical, biological, physical injury and psychosocial factors like depression and stress. As a result, these factors lead to poor health of nurses. Nurses can provide quality care to patients if they are healthy and hold good quality of life. Therefore, it is necessary to pay attention to the general health and quality of life of nurses.

Studies about the HRQoL of nurses in Pakistan are neglected. Therefore, in this study nurses’ health-related quality of life was of particular interest. The aim of this study was to assess nurses’ health-related quality of life in view to physical and psychological health.

## METHODS

This cross-sectional analytical study was conducted in two tertiary care hospitals of Karachi. A total of 154 nurses were included in this study. Written informed consent was taken from all participants and permission was also obtained from the Institutional Review Board (IRB) (IRB-810/DUHS/Approval/2016/354) (10^th^ December, 2016) of Dow University of Health Sciences to conduct the study. Data were completed from May to June 2017. In order to assess nurses’ health-related quality of life, two questionnaires were used Short Form-26 (SF-26) for physical health and PHQ-9 for psychological health of the nurses. The Patient Health Questionnaire-9 (PHQ-9) is a brief 9-item questionnaire designed to identify major depressive disorders and widely used in different population and studies conducted at clinical side as a screening and diagnostic instrument.^14^ Each of the 9 items has scores from 0 to 3, from “not at all” to “nearly every day” respectively. Study participant who score less than less than 5 on PHQ-9 scale was considered as minimal or no depression whereas score ≥ 5 described participant had mild, moderate and severe depression.

SF-26 is a brief 26-items questionnaire form designed to assess the physical health of nurses. The questionnaire form consists of five domains pain, Role limitation due to physical health, Energy/Fatigue, General Health and Physical functioning with two, four, four, five and ten numbers of items in each domain respectively. Each domain of SF-26 consists of dissimilar response with different value and categories. Physical functioning includes category 1,2,3 with response 0, 50 &100, Role limitation due to physical health include category 1, 2 with values 0 &100, Energy/Fatigue with categories (1-6) for response of 100, 80, 60, 40, 20&0, and 0, 20, 40, 60, 80&100 respectively. Whereas, Pain includes categories 1-6 and 1-5 for value 100, 80, 60, 40, 20 &0 and 100, 75, 50, 25 & 0 respectively and General health categories 1-5 for response of 100, 75, 50, 25 & 0 and 0, 25, 50, 75, 100 respectively. Data were analyzed via SPSS version 21.0. Categorical variables were presented through frequencies and percentages whereas means and standard deviations were calculated for quantitative variables. Two samples independent t-test and Analysis of Variance (ANOVA) were performed for identifying any significant mean difference for all five domains of SF-26 with different demographic characteristics. Post-hoc test for pair wise comparison was applied for domains that showed significant difference in ANOVA. T-test was again used to seek whether nurses’ physical health varied with psychological health. P-value ≤ 0.05 was considered as significant.

## RESULTS

In this study the proportion of male participants is higher than female. Most of the participants were graduate (42.21%) and married (56.49%). The registered nurses were highlighted among other designations of the study participants (67.53%). The proportion of the participants working in rotation and fixed duties was 50% for each. There were 55.84% of nurses working as a contractual basis and monthly income of the two-thirds of the participant’s was equal to Rs. 21000-40000/month. Table-I which also depicts mean scores in all five domains of SF-26 with respect to demographic characteristics of the participants. Mean scores of all domains for males were found to be higher than females. The mean scores of physical function and role limitation domains were higher for nurses who married, working fixe duties and working as an assistant head nurse and had income more than Rs. 60000. Mean scores of pain domains were observed greater for nurses who unmarried, working fixed duties, working as a head nurse and earning less than Rs. 20000. No notable mean difference of all SF-26 domains was reported except Role limitation due to physical health (RP) between nurses working in government sector or contractual basis. For energy/fatigue domain higher mean scores were observed for nurses who married, working rotational duties, performing their duties as a registered nurse and earning between 21000 to 40000. General health mean scores were higher for unmarried nurses, had lesser income and working in rotational duties environment. ANOVA and T-test confirmed that energy/fatigue domain differed significantly with level of education, duty shift and monthly income with p-values 0.025, 0.001 and 0.006 respectively. Means scores of the general health domain also varied significantly among different level of monthly income of the nurses (p-value=0.004).

**Table-I:** Demographic Characteristics of study participants and SF-26 Scores.

		N (%)	Mean (SD)				

			PF	RP	P	E& F	GH
Gender	Male	86 (55.84)	54.82 (27.55)	33.72 (46.61)	72.79 (20.93)	53.31 (8.66)	67.49 (10.04)
	Female	68 (44.16)	46. 47 (27.22)	22.05 (40.64)	70.51 (22.04)	53. 89 (9.95)	64.88 (8.67)
	P-value		0.062	0.105	0.514	0.698	0.092
Level of Education	Matric	18 (11.69)	51.94 (26.97)	29.16 (45.57)	74.02 (22.26)	54.16^abcdf^ (9.43)	63.65 (7.93)
	Intermediate	43 (27.92)	48.95 (25.36)	27.32 (43.92)	67.79 (21.71)	56.97^a^ (8.73)	66.76 (9.06)
	Graduate	65 (42.21)	50.53 (29.96)	25.76 (43.06)	73.23 (20.82)	52.15^bde^ (8.79)	67.30 (10.05)
	Post-graduate	28 (18.18)	55.35 (26.8)	36.60 (48.34)	73.12 (21.99)	51.25^cef^ (9.77)	65.17 (9.90)
	P-value†		0.812	0.752	0.555	0.025*	0.461
Marital Status	Single	67 (43.51)	49.70 (30.1)	26.49 (44.14)	71.86 (21.54)	52.68 (10.27)	67.78 (9.55)
	Married	87(56.49)	52.24 (25.66)	30.17 (44.64)	71.72 (21.39)	54.25 (8.33)	65.22 (9.39)
	P-value		0.573	0.611	0.968	0.298	0.099
Job Nature	Government	68(44.16)	51.69 (27.48)	34.19 (46.90)	70.33 (21.12)	53.67 (9.52)	65.56 (9.62)
	Contract	86 (55.84)	50.69 (27.89)	24.12 (41.91)	72.93 (21.65)	53.48 (9.04)	66.95 (9.45)
	P-value		0.825	0.168	0.455	0.901	0.369
Duty shift	Fixed	77 (50)	52.53 (29.33)	31.16 (45.55)	75.06 (20.43)	51.23 (7.528)	66.01 (10.58)
	Rotation	77 (50)	49.74 (25.94)	25.97 (43.19)	68.50 (21.94)	55.90 (10.18)	66.66 (8.38)
	P-value		0.523	0.469	0.057	0.001*	0.674
Designation	Head nurse	21 (13.64)	48.80 (26.73)	29.76 (45.83)	75.00 (19.96)	49.28 (7.62)	66.07 (7.36)
	Assistant Head nurse	13 (8.44)	63.84 (30.42)	38.46 (50.63)	67.30 (28.03)	52.69 (9.04)	65.06 (10.42)
	Registered nurse	103(66.89)	49.36 (25.84)	25.00 (42.15)	71.65 (21.01)	54.07 (9.41)	66.74 (9.76)
	Others	17 (11.03)	55.00 (35.79)	41.17 (50.72)	72.05 (21.03)	52.64 (9.03)	65.19 (10.30)
	P-value†		0.303	0.443	0.792	0.093	0.879
Monthly Income (Rupees)	< 20000	17 (11.03)	52.05 (37.91)	42.64 (49.81)	82.50 (23.61)	49.70^abc^ (7.59)	70.83^ab^ (7.65)
	21000-40000	104 (67.53)	49.27 (25.91)	22.59 (40.85)	70.62 (20.82)	55.33^ad^(9.22)	67.02^ac^ (9.50)
	41000-60000	23 (14.94)	53.19 (26.32)	35.86 (48.16)	66.95 (20.55)	49.34^be^(8.70)	63.22^bcd^ (8.76)
	> 60000	10 (6.50)	62.50 (28.98)	50.00 (52.70)	76.75 (21.70)	51.50^cde^ (8.51)	58.75^d^ (9.09)
	P-value†		0.495	0.086	0.097	0.006*	0.004*

†: P-values from Analysis of Variance (ANOVA),

* : P-value significant at 0.05 level of significance.

The superscripts show the pair-wise significance. The different alphabets show statistical significance.

According to the Patient Health Questionnaire (PHQ-9), 69.48% participants were found as depressive. Table-II shows physical (SF-26 domains) and psychological (depression) health status of the participants is shown in Table-II. Mean±SE of each domain of SF-26 scale with 95% CI are also reported in Table-II. Among depressive participants the highest mean score was obtained for pain 68.6±20.43 whereas the lowest recorded for role limitation due to physical health 21.96±40.44. However, among non-depressive nurses highest mean score was recorded for pain 79.04±21.97 and lowest for role limitation due to physical health 43.62±49.30. Notable mean differences between depressed and non-depressed participants were obtained for three domains physical functioning, role limitation due to physical health and pains. Two-sample independent t-test also confirmed that mean scores of domain physical health (p-value 0.045), role limitation due physical health (pvalue 0.01) and pain (p-value 0.005).

**Table-II:** SF-26 Scores and Depression status of the study participants.

SF-26 Domains	Depression	Mean	S.E	Mean difference	9.5% C.I.	P-value
Physical Functioning	No Depression (n=47)	57.87	4.76	9.69	0.236---19.152	0.045*
	Depression (n=107)	48.18	2.38			
Role limitation due to Physical health	No Depression (n=47)	43.62	7.19	21.65	5.344---37.964	0.01*
	Depression (n=107)	21.96	3.90			
Pain	No Depression (n=47)	79.04	3.20	10.44	3.215---17.673	0.005*
	Depression (n=107)	68.6	1.97			
Energy& Fatigue	No Depression (n=47)	52.77	1.47	-1.16	-4.355---2.037	0.475
	Depression (n=107)	53.93	0.85			
General Health	No Depression (n=47)	66.58	1.23	0.34	-2.962---3.642	0.839
	Depression (n=107)	66.24	0.96			

* : P-value significant at 0.05 level of significance (t-test).

## DISCUSSION

The finding of this study revealed that there no relationship of male and female gender with all five physical domains including physical functioning, Role limitation due physical health, pain, energy and fatigue and general health with p-values 0.62, 0.105, 0.514, 0.698 and 0.092 respectively. This may be due to an equal distribution of tasks among nurses in clinical areas without gender discrimination. These finding were supported by the study conducted in Iran in 2016.^15^ This study showed that the level of education is significantly associated with energy and fatigue level of nursing. It shows that education level seems to have an impact on energy and fatigue level of nurses. These findings were similar to studies conducted in Saudi Arabia^16^ and Ethiopia^17^ in 2014 and 2017 respectively and Iranian study in 2014.^18^ However, study accomplished in Iraq 2017^19^ showed contrary finding.

Our study revealed that the duty shift is significantly associated with energy and fatigue level of nurses. The reason for this association could be that nurses are working in the morning, evening and night duties. Rotational duties can cause a disturbance in sleep pattern and irregular circadian rhythm, which can impact on their health. Nursing profession is demanding both mental as well as physically and it’s requiring energy to cope with daily requirement at clinical settings. Monthly income was also found significantly associated with general health and energy and fatigue level of nurses. It highlighted that nurses’ physical health have a strong association with a monthly income of nurses. The reasons may be due nurses are busy with patients 24/7, so monthly income could effect on energy/fatigue level and general. These findings were supported by the study conducted in Ethiopia in 2017^17^ and study conducted in Iran (2016)^15^ while, Iranian study in 2012^20^ did not report similar results.

In our study few socio-demographic variables like marital status, job nature, and designation were not found to be significantly associated with physical health. These findings may be due to busier schedule of nurses in morning duties, which is significantly associated with physical health. Moradi et al.^18^ reported a similar finding in the study completed in Iran. However, there are research studies^16^,^19^,^21^,^22^ in the literature that showed a significant association between above-mentioned demographic variables with physical health.

We found that some of the physical health domains’ score differ significantly with mental health, these domains include Physical functioning, role limitation due to physical health and pain. Nurses require both physical and psychological fitness for their daily activities. Any problem in physical and psychological health may linked to each other. Konstantinou and Efstathiou^23^ also reported the similar association of the physical health domain with mental health.

Means score of Pain domain was found to be highest among both depressive and non-depressive participants. This may be due to nurse’s busy schedule in their clinical areas; either they were depressive or non-depressive. However, studies conducted in Cyprus^23^ and Brazil 2010^24^ have reported highes mean scores for physical functioning. In our study mean score of Role limitation due to physical health was lowest for both depressed and non-depressed participants. These findings were in contrast with the study completed in Brazil 2010^24^ and the study conducted in Iran 2018.^25^

## CONCLUSION

Health related quality of life differs in comparison of physical health domain with depressive and non-depressive nurses. Only energy/fatigue domain was significantly associated with level of education, duty shift and monthly income of nurses.

### Authors’ Contribution:

**AA:** Conceived Idea, Designed Research Methodology, Manuscript Writing, is responsible for integrity of research.

**AR:** Statistical Analysis, interpreted data and Manuscript proof reading.

**SN:** Data Collection and compilation, Literature Review, Manuscript drafting.

## References

[1] Mooney A (2007). Quality of life:questionnaires and questions. J Health Commun.

[2] Miguel RS, Lopez-Gonzalez AM, Sanchez-Iriso E, Mar J, Cabases JM (2008). Measuring health-related quality of life in drug clinical trials:is it given due importance?. Pharm World Sci.

[3] Ismail A, Mohamed CN (2011). A Comparative Study of Health-Related Quality of Life (HRQoL) of School teachers from the Largest Primary and Secondary Schools in urban Shah Alam Malaysia:A cross sectional study. Int Proc Econ Dev Res.

[4] Beck CA, Shah S (2012). Research on health-related quality of life and cardiac conditions. Home Healthc Now.

[5] Gillen S (2014). Unhealthy lifestyles adversely affect nurses'ability to deliver quality care. Nurs Stand.

[6] Blake H, Lee S (2007). Health of community nurses:A case for workplace wellness schemes. Br J Community Nurs.

[7] Kemppainen V, Tossavainen K, Turunen H (2013). Nurses'roles in health promotion practice:an integrative review. Health Promot Int.

[8] Wu S, Li H, Tian J, Zhu W, Li J, Wang X (2011). Health-related quality of life and its main related factors among nurses in China. Ind Health.

[9] Oyama Y, Fukahori H (2015). A literature review of factors related to hospital nurses'health-related quality of life. J Nurs Manag.

[10] Wilkins K, Shields M (2008). Correlates of medication error in hospitals. Health Reports.

[11] Marcus M, Yasamy MT, Ommeren MV, Chisholm D, Saxena S, WHO (2014). WHO Department of Mental Health and Substance Abuse. Depression A Global Public Health Concern.

[12] WHO. Fact Sheets (2016). Depression 2016.

[13] Sahoo S, Khess CR (2010). Prevalence of depression, anxiety, and stress among young male adults in India:a dimensional and categorical diagnoses-based study. J Nerv Ment Dis.

[14] Kroenke K, Spitzer R, Williams J (2001). The PHQ-9:Validity of a brief depression severity measure. Gen Intern Med.

[15] Ghassemi M, Tavafian SS, Heydarnia A (2016). Socio-Demographic Characteristics and Quality of Life of Nurses suffering from Chronic Non-specific Low Back Pain. Int J Musculoskeletal Pain Prev.

[16] Megahed M (2014). Health-related quality of life among students at King Khalid University–Mohail Asser. Int J Nurs Stud.

[17] Kelbiso L, Belay A, Woldie M (2017). Determinants of Quality of Work Life among Nurses Working in Hawassa Town Public Health Facilities, South Ethiopia:A Cross-Sectional Study. Nurs Res Pract.

[18] Moradi T, Maghaminejad F, Azizi-Fini I (2014). Quality of working life of nurses and its related factors. Nurs Midwifery Stud.

[19] Al-Ameri MH (2017). Night Shift and its Impact upon the Quality of Life of Nurses Working at the Teaching Hospitals of the Medical City Complex in Baghdad City, Iraq. J Nurs Care.

[20] Gholami A, Farsi M, Hashemi Z, Lotfabadi P (2013). Quality of life in nurses working in Neyshabur hospitals. Thrita.

[21] Ioannou P, Katsikavali V, Galanis P, Velonakis E, Papadatou D, Sourtzi P (2015). Impact of Job satisfaction on Greek nurses'Health-related Quality of life. Safety and health at work.

[22] Omrani Z, Talebi E (2018). Quality of Life of Nurses and Related Factors. Int J Epidemiol.

[23] Konstantinou MS, AndriEfstathiou RN, Georgios Charalambous MD (2018). Assessing the Health-Related Quality of Life of Nurses in the Public Sector of Cyprus. Int J Caring Sci.

[24] Silva AA, Souza JM, Borges FN, Fischer FM (2010). Health-related quality of life and working conditions among nursing providers. Revista de SaudePublica.

[25] Omrani Z, Talebi E (2018). Quality of Life of Nurses and Related Factors. Int J Epidemiol Res.

